# Shunt assistant device deception due to pseudovertical posturing

**DOI:** 10.1186/2045-8118-12-S1-P10

**Published:** 2015-09-18

**Authors:** Claudia Craven, Neekhil A Patel, Hasan Asif, Aswin Chari, Edward W Dyson, Samir A Matloob, Patricia Haylock-Vize, Simon D Thompson, Syed N Shah, Andrew R Stevens, Tarek Mostafa, Huan Wee Chan, Jinendra Ekanayake, Ahmed K Toma, Laurence D Watkins

**Affiliations:** 1 Victor Horsley Department of Neurosurgery, National Hospital for Neurology & Neurosurgery, Queen Square, London, UK

## Introduction

The ever present need to balance over drainage with under drainage in hydrocephalus has required innovations including adjustable valves with antigravity devices. These are activated in the vertical position to prevent siphoning. We describe a group of patients who presented with unexplained under drainage caused by activation of antigravity shunt components produced by peculiar head/body position.

## Methods

Single centre case series of hydrocephalus patients, treated with ventriculo-peritoneal shunt insertion. These patients presented with clinical and radiological under drainge syndrome. Medical notes were reviewed for clinical picture and outcome. Radiological studies were reviewed assessing shunt placement and ventricular size.

## Results

Four patients presented with clinical and radiological under drainage syndrome. A consistent posturing of long term hyper-flexion of the neck whilst lying supine was observed. All patients had similar shunt construct (adjustable Miethke proGAV valve and shunt assistant antigravity component). In each of those patients a hypothesis was formulated that neck flexion was activating the shunt assistance antigravity component in supine position. All patients underwent shunt revision surgery removing the shunt assistant device from the cranium and adding an antigravity component to the shunt system at the chest. All patients had clinical and radiological improvement.

## Conclusions

The combination of raised ICP when supine and a resistant shunt assistant could be blamed for worsening hydrocephalus. In bedridden hydrocephalus patients with a shunt assistant, consider the possibility of shunt deception due to abnormal neck positioning. In these patients, antigravity devices should be placed at the chest.

